# Thermal modal analysis of hypersonic composite wing on transient aerodynamic heating

**DOI:** 10.1038/s41598-024-61900-y

**Published:** 2024-05-14

**Authors:** Kangjie Wang, Junli Wang, Zhiyuan Liu, Sheng Zhang, Baosheng Zhang, Wenyong Quan

**Affiliations:** 1 School of Mechanical Engineering, Shaanxi University of Technology, Hanzhong, 723001 Shaanxi China; 2 Shaanxi Key Laboratory of Industrial Automation, Hanzhong, 723001 Shaanxi China

**Keywords:** Hypersonic, Composite wing, Aerodynamic heating, CFD/CSD, Mechanical engineering, Aerospace engineering, Composites, Fluid dynamics

## Abstract

Considering the influence of thermal stress and material property variations, this study employs the Navier–Stokes equations and Fourier heat conduction law to establish a semi-implicit time-domain numerical analysis method for hypersonic aerothermal-structural coupling. Study the temporal variation pattern of different regions of the composite material wing under aerodynamic heating. Using the obtained transient temperature field of the wing, the thermal modal of the wing at different time points is calculated using the finite element method. Additionally, it conducts an analysis and discussion on the factors influencing the thermal modal. Composites can be effectively utilized as thermal protection materials for aircraft. During the aerodynamic heating process, the leading edge temperature reaches thermal equilibrium first, followed by the trailing edge, and the belly plate experiences a slower thermal response. Temperature rise significantly affects higher-order modes, with the change in material properties during the early stages of heating being the dominant factor. This leads to a faster decrease in natural frequency. As heat conduction progresses, the influencing factors of thermal stresses gradually increase, and the natural frequency decreases slowly or even rises.

## Introduction

With the rapid advancement of modern aerospace technology, aircraft are progressing towards higher Mach numbers, leading to a more pronounced issue of aerodynamic heating^1^. The aerodynamic heating, generated by the friction between the aircraft and the air during high-speed flight, causes a sharp increase in wing surface temperature. This temperature rise significantly affects the distribution of the inherent frequencies and structural stiffness matrices of materials, resulting in changes in the thermal modes of the aircraft. Therefore, the thermal/structural problem is a crucial consideration in the design of hypersonic aircraft. As aerodynamic tailoring technology continues to mature, an increasing number of aircraft components are composed of composite materials. Especially in critical components such as high-strength thin-walled structures and skin subjected to harsh flight environments, the demand for high-performance composite materials is gradually growing^2^. Compared to titanium alloy materials, composite materials exhibit characteristics such as lightweight, strong designability, and higher thermal conductivity, making them excellent candidate materials for thermal components in aerospace applications^3^. Consequently, understanding the behavior and mechanisms of the aerothermal effects and thermal modes of composite material components becomes essential.

In the high-speed operational state, aircraft endure severe aerodynamic and thermal loads, operating in harsh flight environments with complex physical processes and highly nonlinear coupling of multiple physical fields. Numerous scholars have conducted extensive research in this area. Prandtl^4^, for the first time, proposed the boundary layer theory for aerothermal solutions, simplifying the N–S equation and providing a foundational analytical method for aerothermal problem-solving. Tran^5^ utilized a unified aerothermoelastic solver for numerical calculations on two-dimensional wings and flat plates. This method considers only one-way coupling, accounting for the influence of temperature on structural deformation but neglecting the impact of structural deformation on aerodynamic heating. Sampaio^6^ also used a one-way coupling approach to calculate the thermal flux distribution of a low aspect ratio wing at different altitudes. Anaïs^7^ based on the CFD method, studied the impact of wing aerothermal effects on aerodynamic efficiency characteristics, revealing that as the wing surface temperature increases, the aerodynamic characteristics deteriorate under all flight conditions. Zhao^8^ pointed out that tightly coupled and adaptive step methods can efficiently solve hypersonic aerothermal problems. On this basis, they investigated the influence of aerothermal effects on structural deformation and aerothermoelastic deformation. Miller^9^ developed a partitioned multiphyics time-stepping algorithm based on a loosely coupled method for high-precision model aerothermal calculations during time progression. Chen^10^ combined the method of adaptive coupled time steps with a tightly coupled analysis strategy, studying the mechanical behavior of hypersonic wing flow-thermal-solid coupling under continuous aerodynamic heating conditions. Zhao^11^ employed the S-A turbulence model to solve the fluid control equations and compared the effects of different boundary conditions on the heat flux density for the airfoil. Huang^12^ proposed a parallel iterative coupled method for computing the thermal environment of the wing surface. Their study indicated a good linear relationship between the Mach number and the stagnation heat flux density. The thermal environment led to a reduction in the wing surface’s natural frequency and stiffness, with a decrease in the elastic modulus being the primary factor. Zhao^13^ employed a hierarchical solving approach to compute the wing surface temperature distribution, thermal modes, and thermal flutter boundaries of low aspect ratio wings in chronological order. Chen^14^ established and solved a coupled aerothermoelastic model for thermal protection panels, indicating that the trends of reference point temperature and normal displacement variation are consistent with the changes in aerodynamic heating heat flux density at that point. Gao^15^ proposed a comprehensive aerothermoelastic analysis process based on multi-field coupling methods for static aerothermoelastic trimming, structural thermal modal analysis, and aerothermoelastic response issues, enabling efficient and precise prediction of stagnation point heat flux density. Chen^16^ proposed a coupled analysis method for aerothermal, radiative heat transfer, and transient heat conduction. The numerical results indicate that the heat flux conducted into the structure has a relatively minor effect on the thermal environment calculation of the leading edge of the lifting surface.

In summary, previous studies have primarily focused on the deterioration of aerodynamic characteristics due to the decrease in aerodynamic heating and elastic modulus at the leading edge of the wing. However, the actual process involves a coupled thermal-fluid-structure interaction. In addition to the intense aerodynamic heating effects, there are also gradual heat conduction and the generation of thermal stress leading to additional geometric stiffness issues. These factors can result in significant temperature gradients across various parts of composite wing structures, making them susceptible to stress concentration caused by thermal stress, which can further lead to the enlargement of internal material cracks and block-like delamination. Previous research has paid relatively little attention to these effects. While considering the thermal-fluid-structure interaction, previous studies have seldom accounted for the decrease in material stiffness due to temperature rise and the additional geometric stiffness induced by thermal stress. In reality, these two mechanisms coexist. In addition, most existing studies focus on the analysis under steady-state conditions, while transient thermal modal analysis, especially considering the variation of aerodynamic loads and heating time during flight, remains insufficiently explored. Therefore, this study adopts the partitioned solution method proposed by predecessors and utilizes the CFD/CSD approach to establish a thermo-fluid–structure coupling model, which is validated for reliability and mesh convergence. The model comprises aerodynamic heating, transient heat conduction, material stiffness, and additional geometric stiffness variations with heating time. Temperatures during aerodynamic heating at the wing leading edge, belly plate, and trailing edge locations are computed. The study particularly investigates the influence of the wing temperature distribution on the thermal modal shapes and frequencies during large-scale transient heating processes and analyzes the effect of stiffness factors on the thermal modal frequencies during heat conduction.

## Methods

### Thermal modal calculation flow

**Figure 1:**
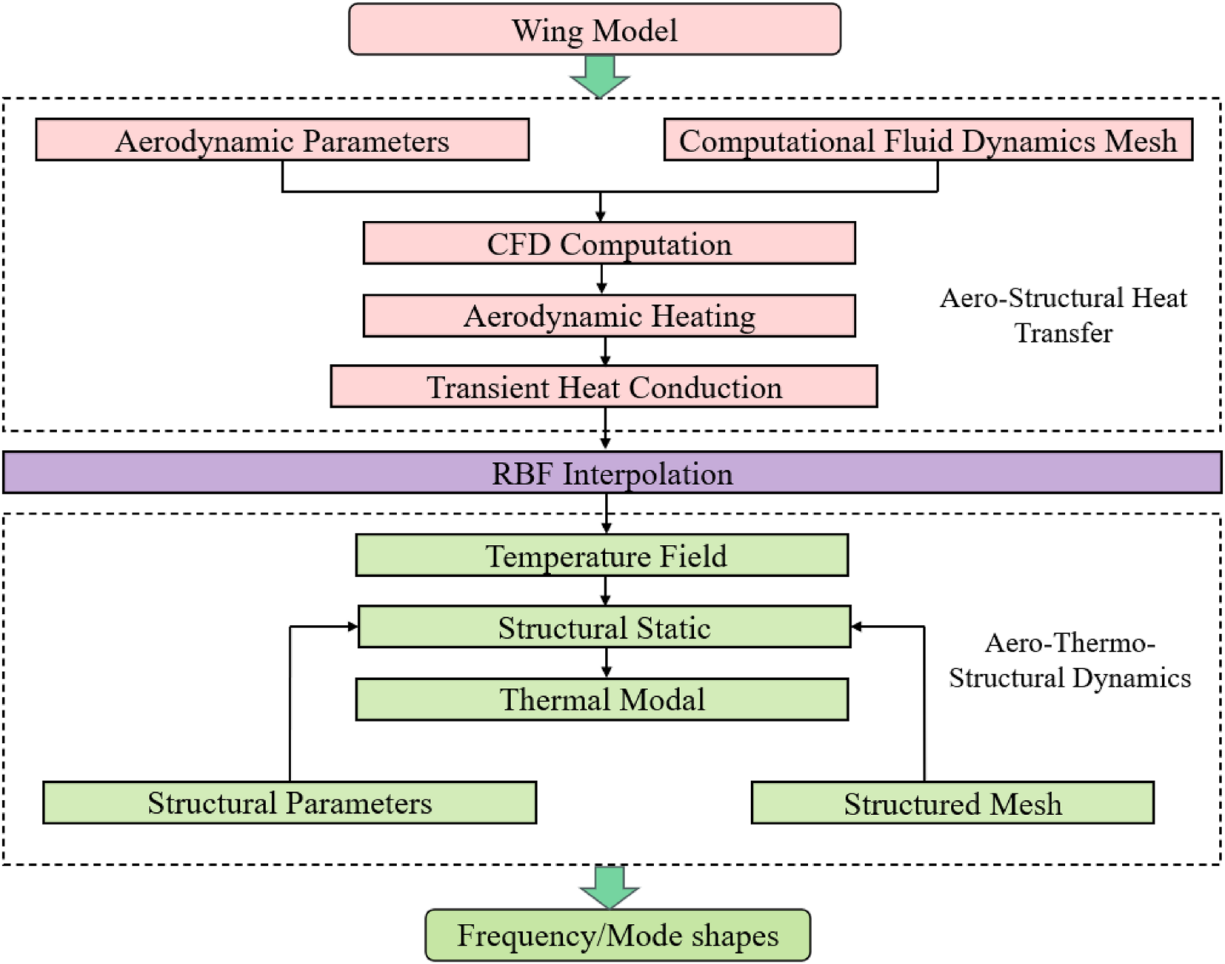
The computational workflow for thermal modal analysis.

During the high hypersonic flight of the aircraft, it is subjected to significant aerodynamic heating effects. In response to the thermal conditions induced by aerodynamics on the wing structure, the coupling relationship between aerodynamics and structure is considered. The Computational Fluid Dynamics (CFD) method is employed for aerothermal calculations. The thermal modal analysis of the wing is then conducted using the load transfer method. The computational process for thermal modal analysis is illustrated in Fig. 1.

The density-based solver calculates the governing equations of fluid flow based on density and velocity, suitable for addressing problems with high Mach numbers and large compressible flow ranges. Therefore, this paper employs the density-based solver in CFD simulations to complete the aerodynamic-structural heat transfer calculation module. Subsequently, the transient temperature field obtained from CFD calculations is converted into equivalent thermal loads and interpolated onto corresponding nodes in CSD using the Radial Basis Function (RBF). Finally, the structural solver is used for the modal shapes and frequencies calculation under transient aerodynamic heating conditions.

### Aerothermal computational method

Disregarding volume forces and internal heat sources, for the aerodynamic heating issue on the wing surface, the governing equations can be expressed using the integral form of the Navier–Stokes control equations in a Cartesian coordinate system^17^.

1 $$\begin{array}{c}\frac{\partial }{\partial t}\underset{\Omega }{\iiint }\mathcal{Q}{\text{d}}V+\underset{\partial\Omega }{\iint }\left({F}_{c}+{F}_{\nu }\right)\cdot n{\text{d}}S=0\end{array}$$

where Q represents the conservation vector; ${F}_{c}$ denotes the convective flux; ${F}_{\nu }$ signifies the viscous flux; ${\text{d}}S$ represents the control volume boundary interface; and $n$ is the unit outward normal vector of the boundary interface.

In the absence of internal heat sources, the heat flux within the solid follows Fourier’s law of heat conduction with respect to temperature^18,19^:

2 $$\begin{array}{c}c\rho \frac{\partial T}{\partial t}=\frac{\partial }{\partial x}\left(\lambda \frac{\partial T}{\partial x}\right)+\frac{\partial }{\partial y}\left(\lambda \frac{\partial T}{\partial y}\right)+\frac{\partial }{\partial z}\left(\lambda \frac{\partial T}{\partial z}\right)\end{array}$$

In the equation, $c$ represents the specific heat capacity; $\rho$ stands for density, and $\lambda$ denotes the thermal conductivity.

In the paper, a semi-implicit^20^ Computational Fluid Dynamics (CFD) method based on the Navier–Stokes equation is employed to calculate the heat transfer coupling in the aerodynamics-structure system. Concerning the governing flow control equations mentioned above, an implicit time-stepping format is applied. The convective flux is discretized using a flux-difference splitting scheme, and the viscous flux is discretized using a central format. To model the turbulent terms, the two-equation shear stress transport (SST) turbulence model proposed by Menter^21^ was used in the computational analysis. Additionally, for hypersonic aerodynamic heating problems, the viscosity coefficient is typically computed using the Sutherland formula^22^, which describes the relationship between the viscosity of an ideal gas and the absolute temperature. This calculation significantly affects the heat flux density and computational accuracy.

The Radial Basis Function (RBF) interpolation method is a multidimensional spatial interpolation technique used for transferring interface information in fluid–structure coupling. It can interpolate data with high accuracy, effectively capturing nonlinear relationships and adapting to complex data distributions. Moreover, it exhibits excellent global approximation performance. In multivariate theory, employing RBF for interpolation calculations has become a highly effective numerical method. Its general form can be expressed as follows^23,24^:

3 $$\begin{array}{c}f\left(x\right)=\sum_{i=1}^{N}{\alpha }_{i}\varphi \left(\Vert x-{x}_{i}\Vert \right)+q\left(x\right)\end{array}$$

In the equation: $f\left(x\right)$ represents the function value at position $x$; ${x}_{i}$ denotes the position of the radial function base points; $\Vert x-{x}_{i}\Vert$ indicates the distance between two points; $N$ is the number of radial function base points; ${\alpha }_{i}$ represents the interpolation coefficients; $\varphi$ is the radial basis function; $q\left(x\right)$ denotes a linear polynomial about the $x$ component.

### Structural thermal modal calculation method

Considering the geometric nonlinearity phenomenon induced by thermal stress, this paper utilizes the Von-Kármán large deformation theory to elucidate the impact of thermal stress on the wing surface of composite materials. The relationship of strain can be expressed as^25^:

$$\varepsilon ={\varepsilon }_{m}+{\varepsilon }_{mb}+{\text{z}}\kappa =$$

4 $$\begin{array}{*{20}c} {\left[ {\begin{array}{*{20}c} {\frac{{\partial u}}{{\partial x}}} \\ {\frac{{\partial v}}{{\partial y}}} \\ {\frac{{\partial u}}{{\partial y}} + \frac{{\partial v}}{{\partial x}}} \\ \end{array} } \right] + \left[ {\begin{array}{*{20}c} {\frac{1}{2}\left( {\frac{{\partial {\mathcalligra{w}}}}{{\partial x}}} \right)^{2} } \\ {\frac{1}{2}\left( {\frac{{\partial {\mathcalligra{w}}}}{{\partial y}}} \right)^{2} } \\ {\frac{{\partial {\mathcalligra{w}}}}{{\partial x}}\frac{{\partial {\mathcalligra{w}}}}{{\partial y}}} \\ \end{array} } \right] + z\left[ {\begin{array}{*{20}c} { - \frac{{\partial ^{2} {\mathcalligra{w}}}}{{\partial x^{2} }}} \\ { - \frac{{\partial ^{2} {\mathcalligra{w}}}}{{\partial y^{2} }}} \\ { - 2\frac{{\partial {\mathcalligra{w}}}}{{\partial x}}\frac{{\partial {\mathcalligra{w}}}}{{\partial y}}} \\ \end{array} } \right]} \\ \end{array}$$

Aerodynamic heating causes a rapid increase in structural temperature. This temperature rise induces changes in the material and geometric stiffness of the structure^12^, subsequently affecting modal shapes and natural frequencies of the structure.

1. Material stiffness.

The increase in temperature induces a decrease in the elastic modulus of the material, resulting in a reduction in the material stiffness of the structure. In a thermal environment, the material stiffness matrix ${K}_{T}^{e}$ for the structure can be expressed as^26^:

5 $$\begin{array}{c}{K}_{T}^{e}=\int {B}^{T}{D}_{T}B\mathrm{d\Omega }\end{array}$$

In the equation: $B$ represents the geometric matrix; ${D}_{T}$ denotes the elastic matrix of the element at elevated temperatures, which is associated with the elastic modulus and Poisson’s ratio, changing with variations in temperature.

(2) Geometric stiffness.

The uneven temperature distribution in a heated structure induces thermal stresses, giving rise to geometric nonlinearity and resulting in additional geometric stiffness matrix ${K}_{S}^{e}$. The geometric stiffness matrix ${K}_{S}^{e}$ for the structure under thermal conditions can be expressed as^26^:

6 $$\begin{array}{c}{K}_{S}^{e}=\int {N}^{T}{S}_{T}N\mathrm{d\Omega }\end{array}$$

In the equation: $N$ is the element shape function matrix; ${S}_{T}$ is the thermal stress matrix.

Vibration theory indicates, that tensile and compressive stresses within a structure alter the system’s regularized stiffness, inducing both ‘hardening’ and ‘softening’ effects that impact the thermal-aeroelasticity of the structure^27^. This phenomenon is particularly pronounced in beam, plate, and shell elements. The stresses induced in the structure by temperature can be simulated by considering additional stiffness in the stiffness matrix, incorporating both the material stiffness matrix ${K}_{T}^{e}$ and the additional stiffness matrix ${K}_{S}^{e}$. The stiffness matrix under thermal conditions ${K}^{e}$ can be expressed as the sum of the material stiffness matrix and the additional stiffness matrix.

7 $$\begin{array}{c}{K}^{e}={K}_{S}^{e}+{K}_{T}^{e}\end{array}$$

The generalized eigenvalue equation for the thermal modes of the structure can be expressed as:

8 $$\begin{array}{c}\left({K}^{e}-{\omega }^{2}\right)\phi =0\end{array}$$

### Method validation

Allan^28^ conducted aerothermal heating experiments on stainless steel round tubes at the NASA Langley Research Center’s high-temperature wind tunnel. This experiment has become a standard case for validating the coupling of flow field and structural heat transfer. In this study, the same stainless steel round tube model as in the experiment was selected. The tube has an outer diameter of 38.1 mm, inner diameter of 25.4 mm, and is made of standard 1Cr18Ni9Ti stainless steel. Its thermodynamic parameters are as follows: density $\rho$ = 8030 kg·m^−3^, specific heat capacity C_s_ = 502.48 J·kg^−1^·K^−1^, and thermal conductivity ${k}_{s}$ = 16.27. The inflow conditions are shown in Table 1.

**Table 1:** Inflow Condition Parameters.

${Ma}_{\infty }$	${T}_{\infty }/K$	${P}_{\infty }/Pa$	${Re}_{\infty }/m$
6.47	241.5	648.1	$1.31\times {10}^{6}$

**Figure 2:**
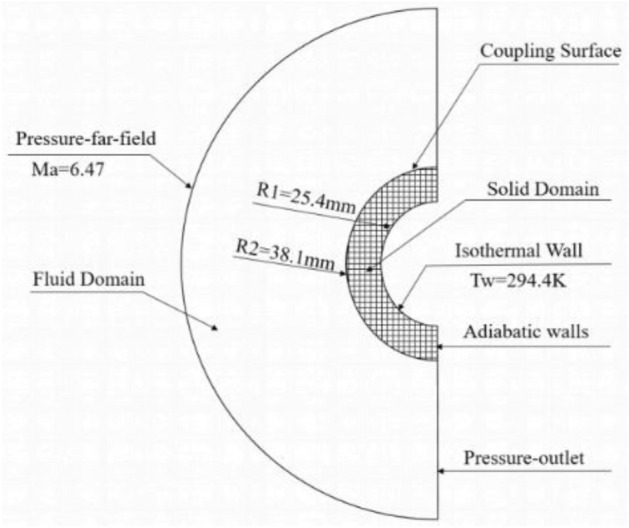
Verification of the model’s boundary conditions.

To conduct high-speed integrated unsteady calculations on the stainless steel circular tube, and to ensure the accuracy of the heat flux density calculation at the wall, the first layer mesh height at the wall is set to $1\times {10}^{-5}$ m. Mesh refinement is applied at the shockwave location, and the boundary conditions are shown in Fig. 2.

In addition, to achieve the same convergence criteria, the explicit standard format requires 10,000 steps, while the implicit AUSM format requires only 4000 steps, significantly saving computational resources. The transient total coupling calculation time in this study is set to 2 s, and the analysis time step is $\Delta t=1\times {10}^{-4}$ s.

**Figure 3:**
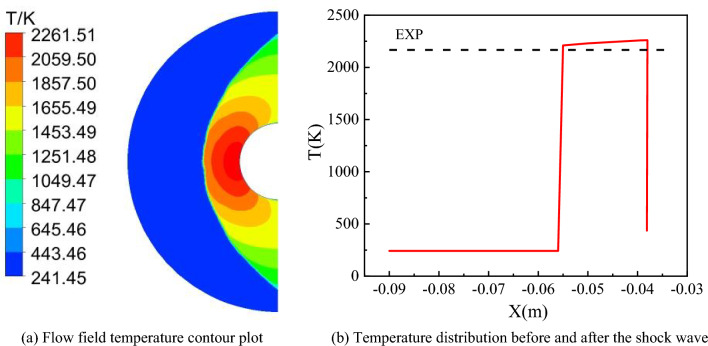
Validation of CFD Calculation Method.

Before conducting aerodynamic heating calculations, the Computational Fluid Dynamics (CFD) method was validated using an isothermal wall boundary condition. Figure 3a shows a temperature contour map of the flow field, indicating that the bow-shaped shock wave ahead of the circular tube is clearly captured. Figure 3b presents the temperature distribution along the x-axis before and after the shock wave. The computed shock wave position is at − 0.055 m, which agrees well with experimental results. The post-shock wave temperature is 2261 K, slightly higher than the experimental value.

**Figure 4:**
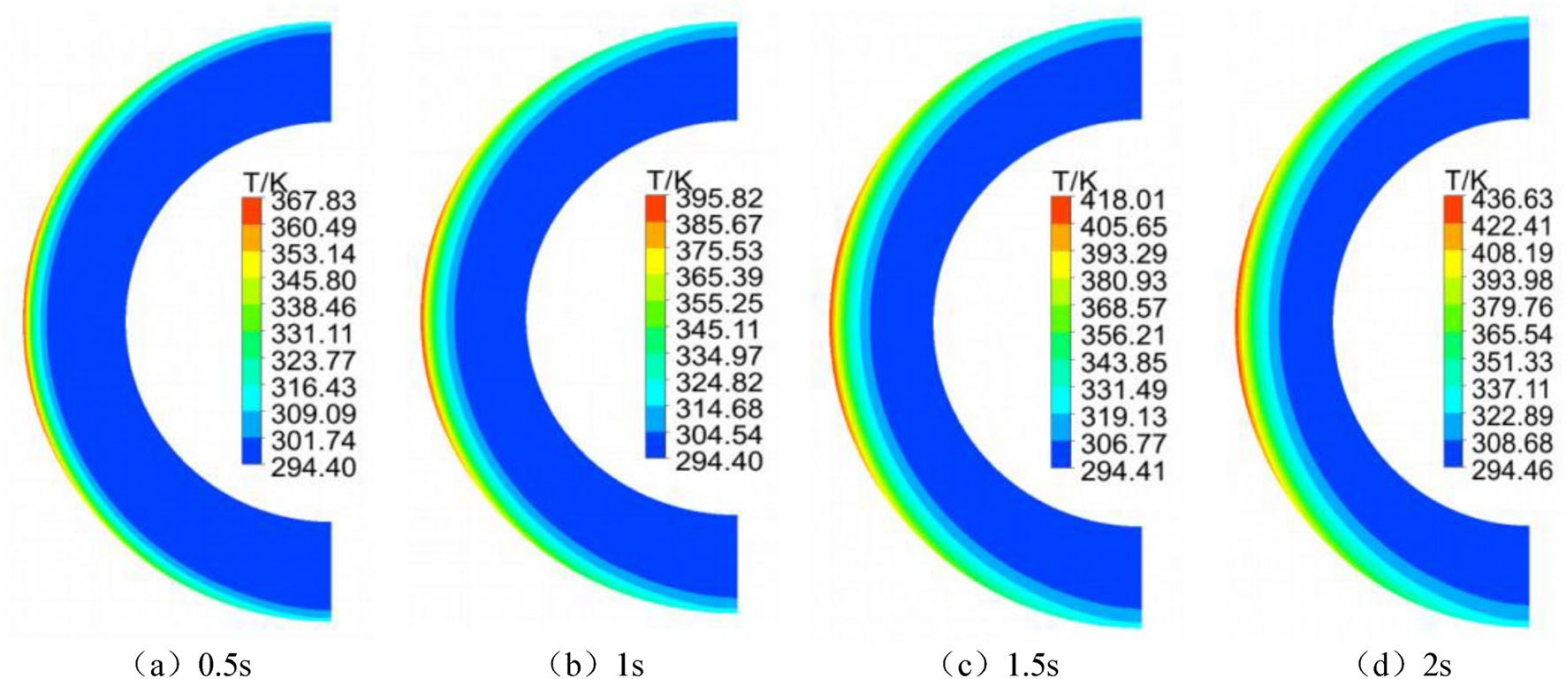
Temperature contour plot of aerodynamic heating for the circular tube over time.

Figure 4 illustrates the transient temperature distributions of the circular tube at different time steps (0.5 s, 1 s, 1.5 s, 2 s). The temperature contour plots reveal that, following aerodynamic heating through the shock wave, the highest temperature occurs at the leading-edge stagnation point. As aero-thermal heating progresses, the stagnation point temperature continues to rise, and the heated region significantly expands. By the 2 s mark, the stagnation point temperature reaches 436.63 K, representing only a 1.1% increase compared to the result in reference^29^. The relative errors remain below 1% at 0.5 s, 1 s, and 1.5 s. The computed heat flux density at the stagnation point in this study is $68.6\times {10}^{5}$  W/m^2^, slightly higher than the experimental value of $67\times {10}^{5}$  W/m^2^, with a relative error of 2.3%. This validation case demonstrates the well-aligned temperature and heat flux density of the circular tube with experimental and literature values, affirming the effectiveness of the computational method in transient flow fields and the reliability of the results.

**Figure 5:**
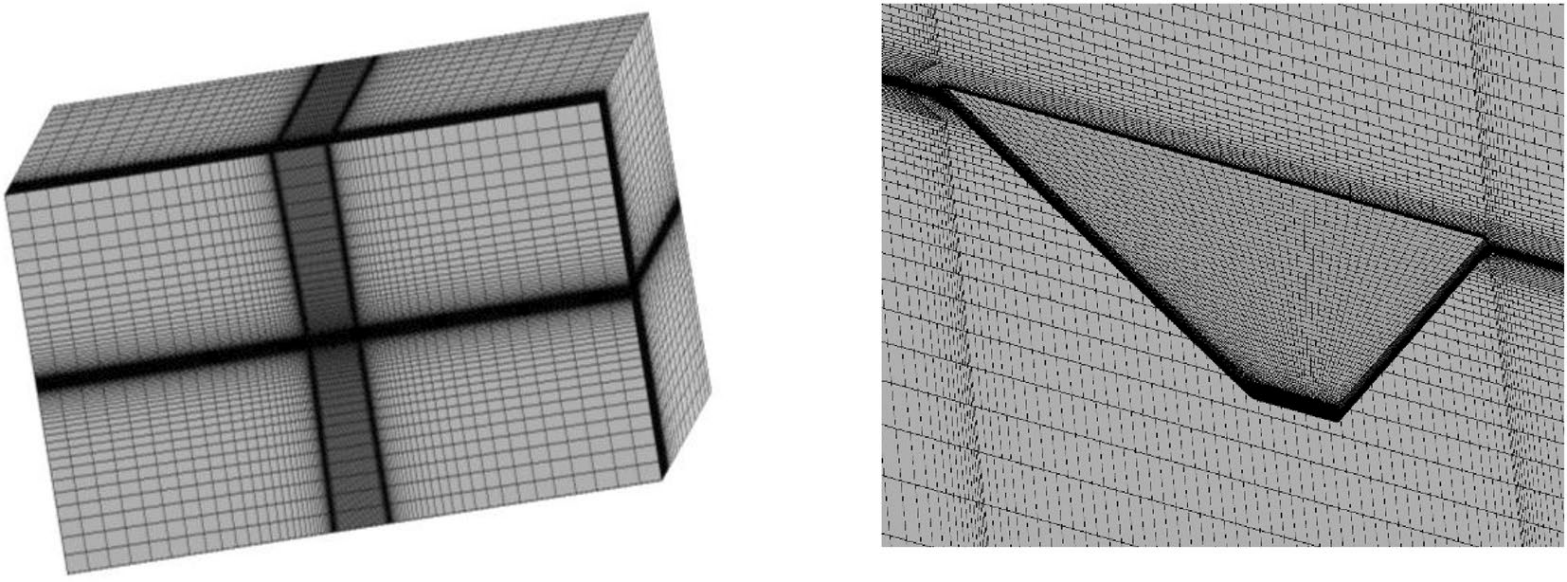
Computational domain and CFD mesh distribution.

**Figure 6:**
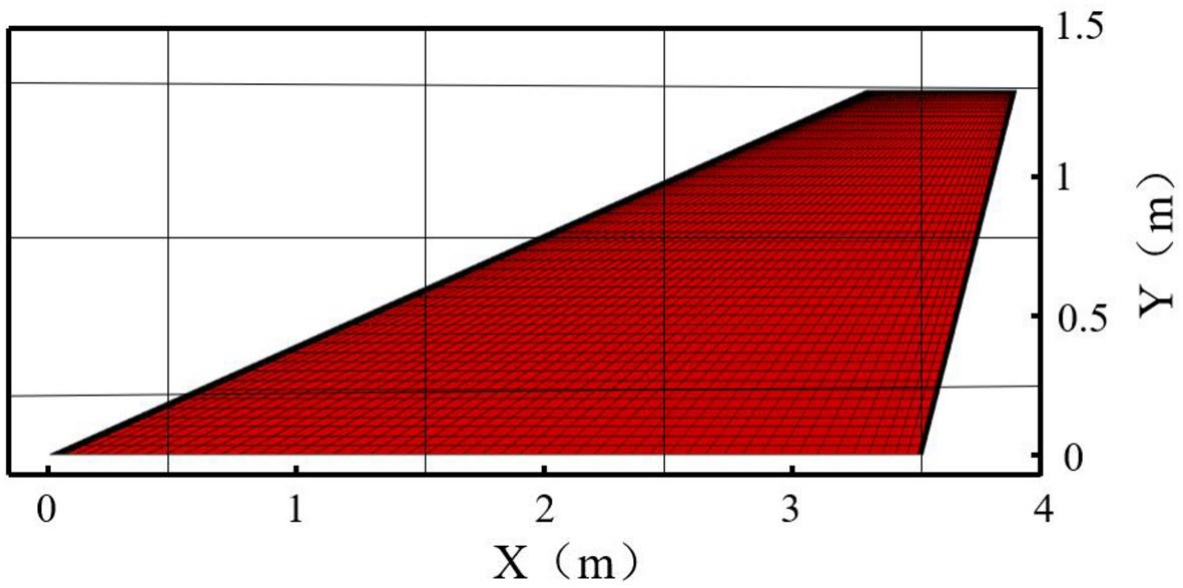
Mesh for CSD calculations.

Based on the characteristics of hypersonic vehicles, a model with a large sweep angle and small aspect ratio was established in this study. Specifically, the geometric parameters of the wing are as follows: root chord length of 3.5 m, tip chord length of 0.6 m, span of 1.25 m, and leading edge sweep angle of 20°. The wing profile adopts a flat plate with rounded leading and trailing edges, with a thickness of 4 × 10^–2^ m. Figure 5 illustrates the distribution of the computational domain and mesh in the CFD simulation, with the first-layer mesh height of 6 × 10^–5^ m and refinement applied to the leading and trailing edges. Figure 6 displays the mesh for structural analysis, where a consistent set of hexahedral mesh elements was employed throughout the computation process to accurately simulate thermal conduction in the structure and mitigate the influence of interpolation on computational accuracy. The total number of mesh elements is approximately 1.26 million. The structural material used is titanium alloy T47, with material properties consistent with those described in reference^30^. The model is simulated at a flight altitude of 15 km, a flight speed of Mach 6, and a cruise angle of attack of 10°, with an initial temperature of 300 K.

**Figure 7:**
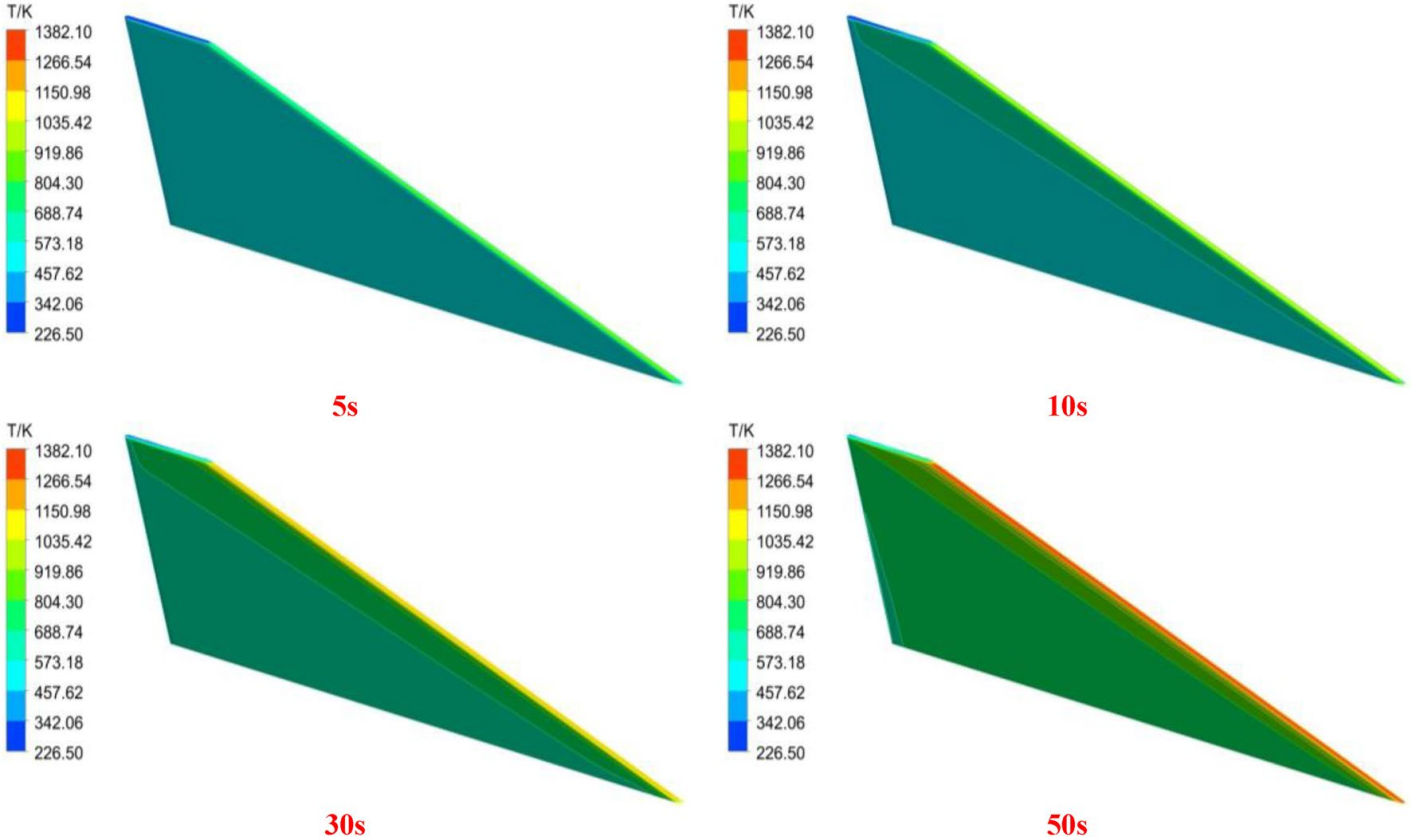
Temperature distribution of the wing surface at different times.

The transient heat conduction process is a critical component for accurately simulating aerodynamic heating analysis. Figure 7a–d depict the temperature distribution on the wing surface at different time instants during the aerodynamic heating process. It can be observed that the temperature is higher and rises rapidly at the leading edge. By the time of 50 s, the temperature at the leading edge has already reached 1380 K, while the overall temperature of the wing is above 600 K. The figures represent the windward surface of the wing. The computational results in this study agree well with those in reference^30^, indicating the accuracy of the transient heat conduction calculation method employed in this paper.

## Numerical model

### Geometry model

**Figure 8:**
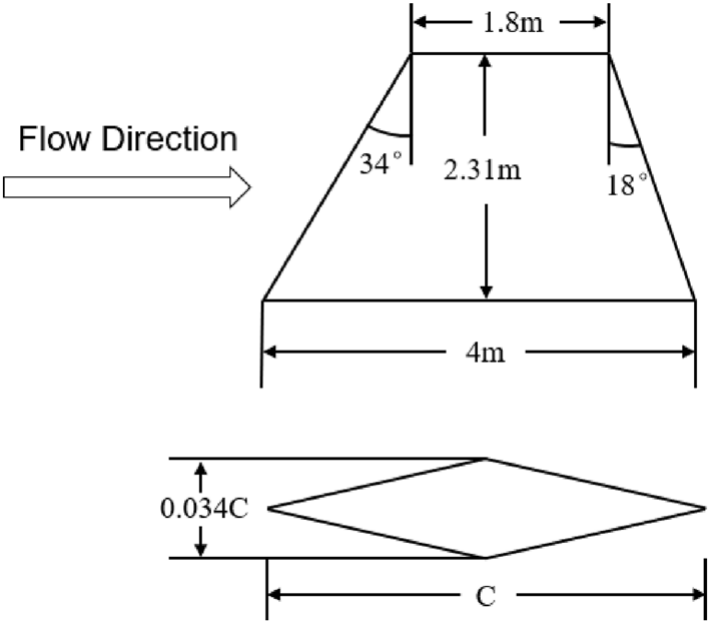
Rhombic wing geometry and dimensions.

This paper analyzes a typical high-hypersonic X-43 aircraft model with a small aspect ratio, featuring a diamond-shaped wing, as shown in Fig. 8. Detailed descriptions of the wing’s outline and geometric dimensions are provided^31^. The root chord length is 4 m, the span is 2.31 m, and the root chord section’s leading and trailing edges are treated using a rounding method with a radius of 8.5 $\times$ 10^–3^ m. The main structure of the wing is constructed from high-temperature-resistant C/SiC composite material, and its thermodynamic parameters^32^ are presented in Table 2.

**Table 2:** Mechanical Properties of C/SiC Composite Materials.

Temperature (K)	Thermal conductivity (W/m K)	Density (kg/m^3^)	Specific heat capacity (J/kg K)	Poisson’s ratio	Elastic Modulus (GPa)	Thermal expansion coefficient 10^–6^ (K^−1^)
300	8	2000	800	0.06	124	0.38
570	8	2000	900	0.06	112	3.09
870	8	2000	1200	0.06	102	3.72
1170	8	2000	1500	0.06	83	3.41
1370	8	2000	1700	0.06	91	4.49
1570	8	2000	1700	0.06	68	3.68
1660	8	2000	1800	0.06	33	4.01

### Mesh partitioning

To ensure the accuracy of the computational results in this study, the mesh convergence of the model was evaluated. The criterion used was to maintain a Y^+^ value of less than 1 and a growth ratio of 1.15. Using ICEM software, five sets of calculation meshes with different densities were generated through refinement. The mesh sizes were 0.83 million, 22 million, 1.5 million, 1.74 million, and 2.11 million, respectively, and the parameters of each mesh set are shown in Table 3.

**Table 3:** Mesh parameters of Calculate model.

Case	Number of mesh (million)	The first layer height (m)	Calculated value (K)
Case1	0.83	5 $\times$ 10^–7^	1563.2
Case2	1.22	5 $\times$ 10^–7^	1587.4
Case3	1.50	5 $\times$ 10^–7^	1596.9
Case4	1.74	5 $\times$ 10^–7^	1605.6
Case5	2.01	5 $\times$ 10^–7^	1611.8

**Figure 9:**
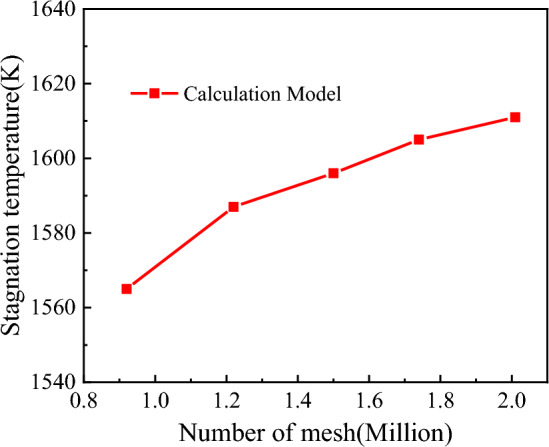
The calculation curve of mesh convergence.

Figure 9 presents the variation of stagnation temperature at different mesh densities. The stagnation temperature monotonically changes with the number of meshes, indicating good convergence of the computational results. This outcome demonstrates the reliability of the mesh refinement and flow field techniques employed in this study.

**Figure 10:**
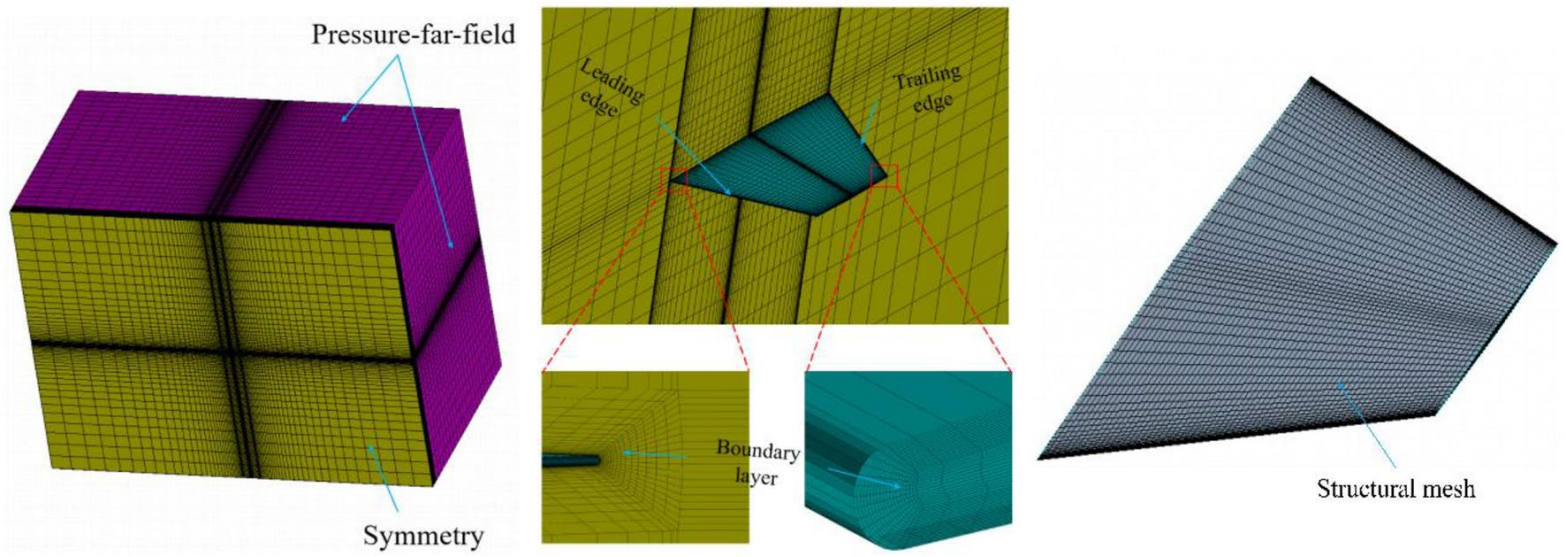
Computational domain and structural mesh partitioning.

In summary, in the computational model of this paper, mesh partitioning follows case 3, ensuring that the first layer height on the wall is less than $5\times {10}^{-7}$ m. The total number of mesh elements is approximately 1.5 million, and the structural mesh for the wing calculation domain is illustrated in Fig. 10. Computational conditions are set for freestream Mach numbers ${M}_{\infty }$ of 5, 5.25, 5.5, and 5.75, at a flight altitude of 15 km, with a freestream static temperature of 216.5 K and static pressure of 12,110 Pa. The angle of attack is set to 0°, and the initial temperature on the wing surface is 300 K for thermal modal analysis. In the computation of the wing’s thermal modal characteristics, the displacement boundary condition is applied, fixing the wing root.

## Calculation results and discussion

### Steady-state temperature field of the wing

**Figure 11:**
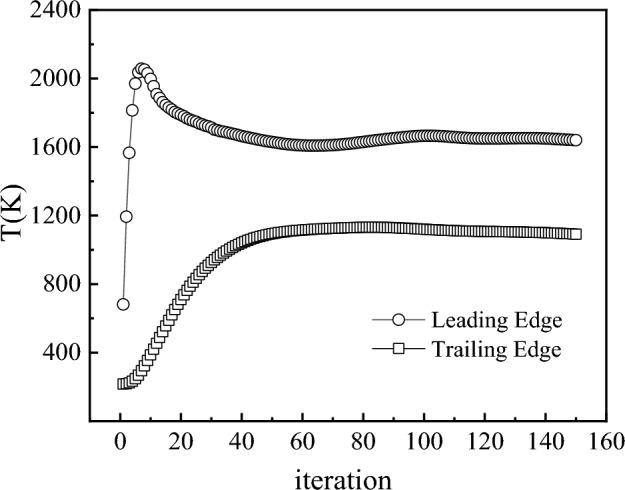
Convergence process of the Leading Edge and Trailing Edge points of the wing.

The surface temperature field of the C/SiC composite material wing was obtained through a steady-state aerothermal-structural coupling calculation method. Figure 11 shows the convergence of temperature at the leading edge point and trailing edge point (y/b = 0.432) during the coupled analysis process. It can be observed from the figure that the temperature at the leading edge point and trailing edge point basically converge after 60 iterations. Here, y/b represents the relative position on the wing profile plane, defined as the ratio of the distance from the wing root to the half-span length of the wing.

**Figure 12:**
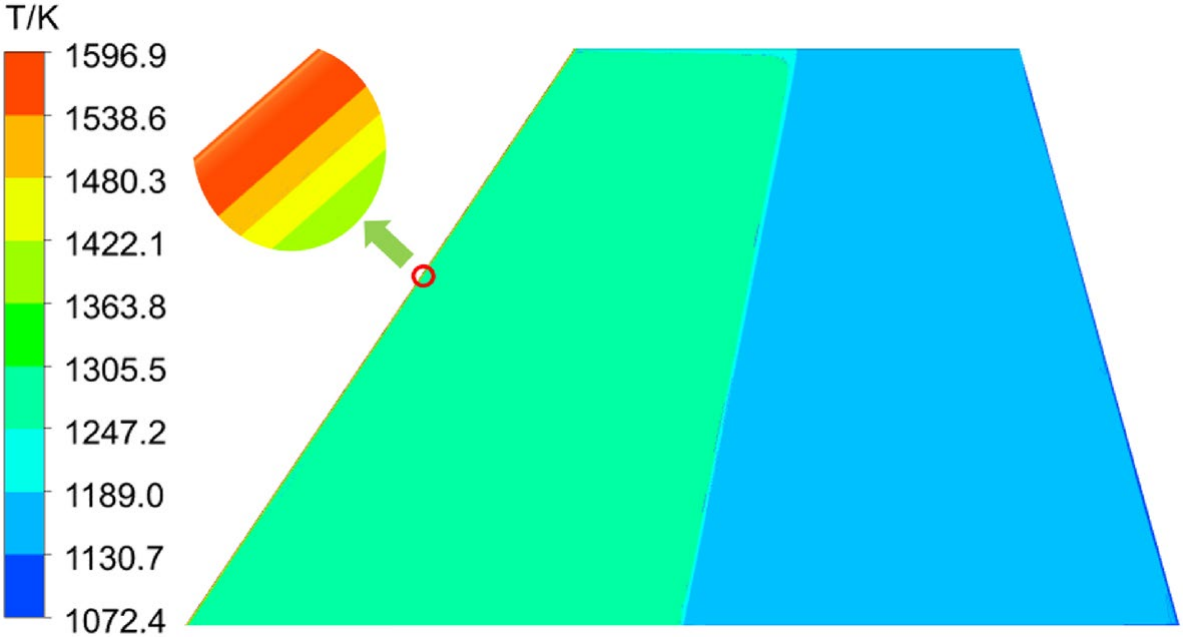
Temperature distribution contour of the C/SiC composite material wing.

Figure 12 illustrates the steady-state temperature distribution map of the wing at Ma = 5. The results indicate the formation of a shockwave at the sharp leading edge of the wing. Following intense shockwave heating, the temperature at the leading edge rises sharply, reaching a maximum of 1600 K. The temperature gradually decreases along the chordwise direction, with the midsection of the wing reaching a temperature of approximately 1300 K. This is attributed to the formation of an expansion wave as the airflow rises in the midsection of the wing, leading to a temperature decrease. The trailing edge of the wing, situated after the expansion wave, experiences a temperature reduction to around 1100 K.

**Figure 13:**
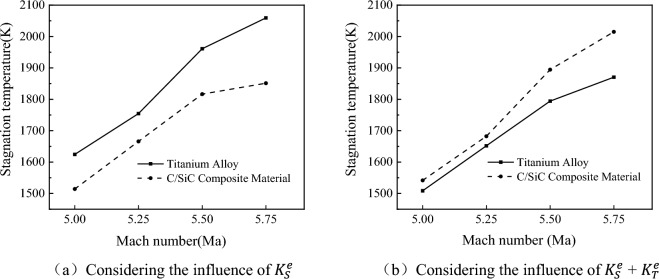
Depicts the variation of stagnation temperature with freestream Mach number.

Figure 13 depicts the variation of stagnation temperature for titanium alloy and composite materials at different freestream Mach numbers: (a) considering only the influence of ${K}_{S}^{e}$, and (b) considering both ${K}_{S}^{e}$ and ${K}_{T}^{e}$. Observing the figure, it can be noted that with an increase in freestream Mach number, the stagnation temperature rises. Without considering the influence of high temperatures, the calculated results are higher for titanium alloys and lower for C/SiC composites. The stagnation temperature has reached the upper limit of titanium alloy usage temperature. From the perspective of thermal safety, it is necessary to use C/SiC composite materials as leading-edge heat-resistant materials to enhance the high-temperature robustness of the wing. When considering the impact of high temperatures on material performance, the stagnation temperature of C/SiC composite material is higher than that of titanium alloy. However, since the upper limit of C/SiC composite material usage is around 2300 K, it can be used under these flight conditions.

**Figure 14:**
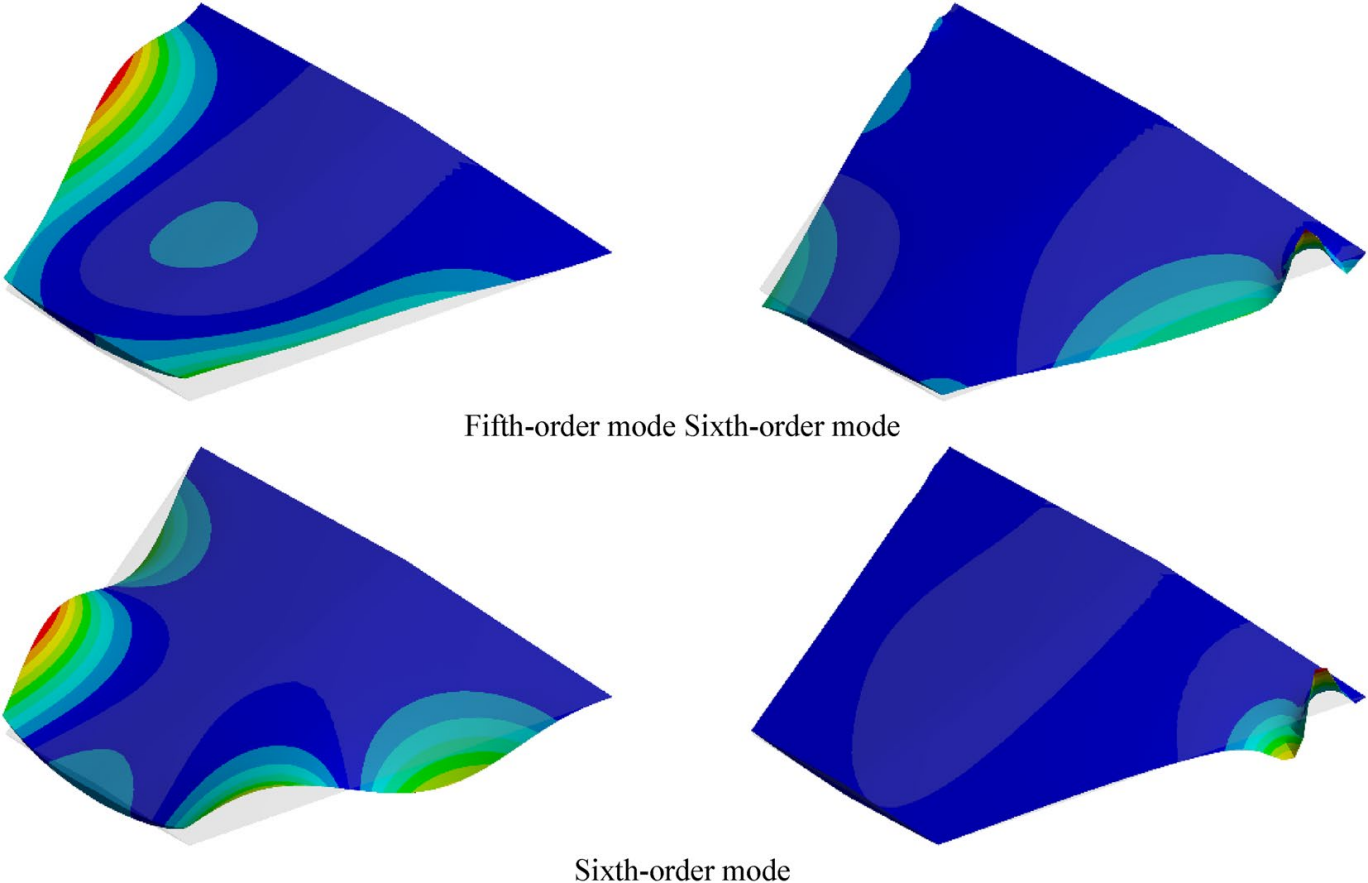
Comparison of high-order mode shapes.

The aerodynamic heating phenomenon has a significant impact on higher-order modes. Figure 14 depicts the mode shape contour plots of these higher-order modes. It can be observed from the figure that significant changes have occurred in the fifth and sixth mode shapes. Specifically, the changes in the fifth mode shape are primarily attributed to the elevated temperature of the wing structure, while the sixth mode shape exhibits characteristics of localized modal behavior. This phenomenon will be further investigated in subsequent discussions.

### Transient heat conduction of the wing

**Figure 15:**
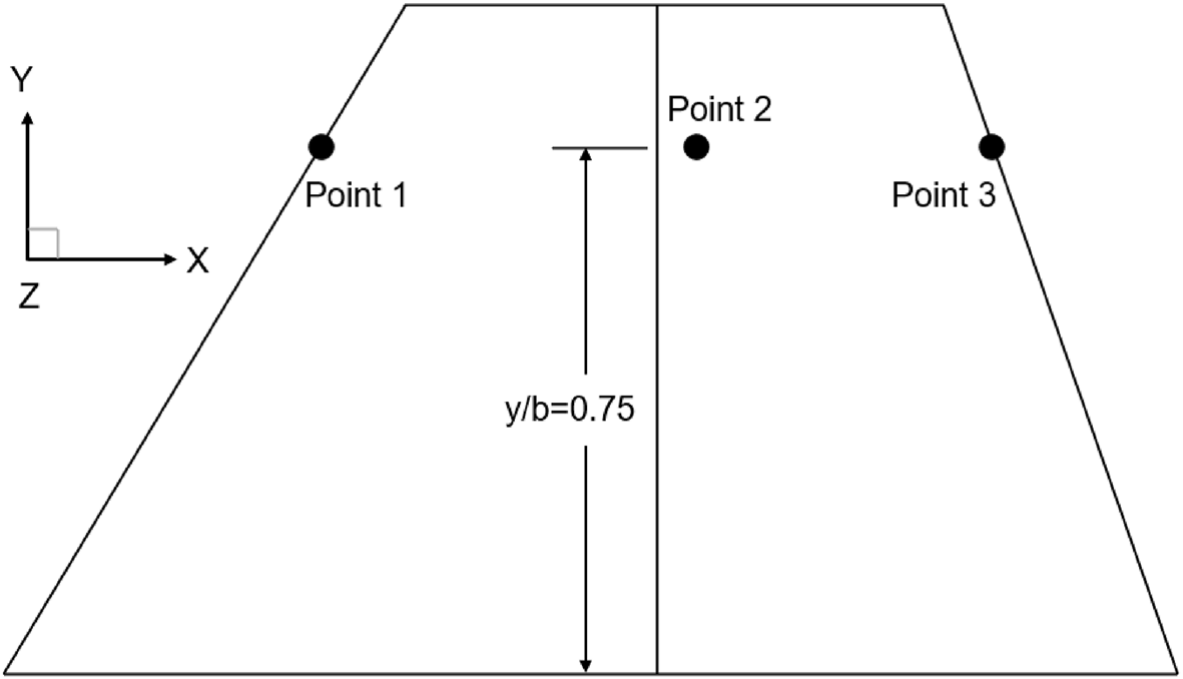
Distribution of temperature monitoring points on the wing.

The temperature of hypersonic aircraft obviously changes with time during the flight, and it is important to analyze the aerothermoelastic variation trend. For a detailed analysis of the transient heat conduction process in the composite wing structure, as shown in Fig. 15, three monitoring points are selected on the wing structure. Points 1–3 are located respectively at the leading edge, belly plate, and trailing edge of the wing at y/b = 0.75 of the wing span.

Transient temperature field calculation of the wing aims to examine the variation of temperatures in different regions of the wing surface over time under the effect of aerodynamic heating. Considering the long-time scale nature of the structural response to aerodynamic heating and to reduce computational burden, this study adopts a segmented approach. The entire aerodynamic heating process is divided into two phases. The first phase spans from 0 to 1 s with a time step of 0.001 s, while the second phase extends from 1 to 50 s with a time step of 0.005 s, The initial temperature of the wing surface is set to 300 K.

**Figure 16:**
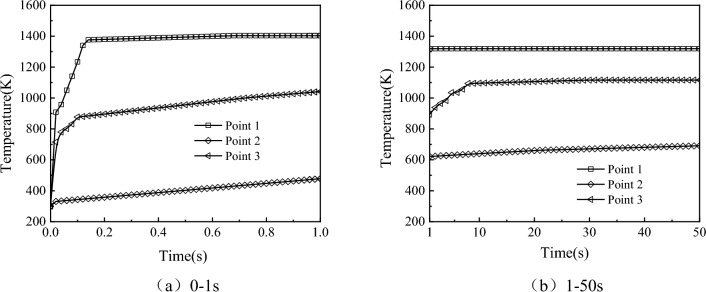
Temperature variation curves of monitoring points over time.

Figure 16 shows the temperature variation curves of the monitoring points over time: (a) illustrates the wing’s thermal environment during the 0–1 s aerodynamic heating, and (b) illustrates the wing’s thermal environment during the 1–50 s aerodynamic heating. From the plot (a), it can be observed that intense aerodynamic heating and slow heat conduction phenomena lead to a rapid temperature rise at the leading edge point. As aerodynamic heating time increases, the leading edge temperature gradually saturates around 0.7 s, with the trailing edge temperature higher than the belly plate temperature. In plot (b), the trailing edge temperature rapidly increases, reaching a thermal equilibrium state around 30 s, while the belly plate undergoes a slow warming process due to the combined effects of trailing-edge heat conduction and expansion waves.

The residual results of the wing surface temperature in the case study indicate convergence. However, the overall wing structure did not reach thermal equilibrium within the 50-s calculation period. Certain regions still exhibit slow heat conduction. This phenomenon is attributed to factors such as the low thermal conductivity of the composite material and the low temperature of the high-altitude environment.

**Figure 17:**
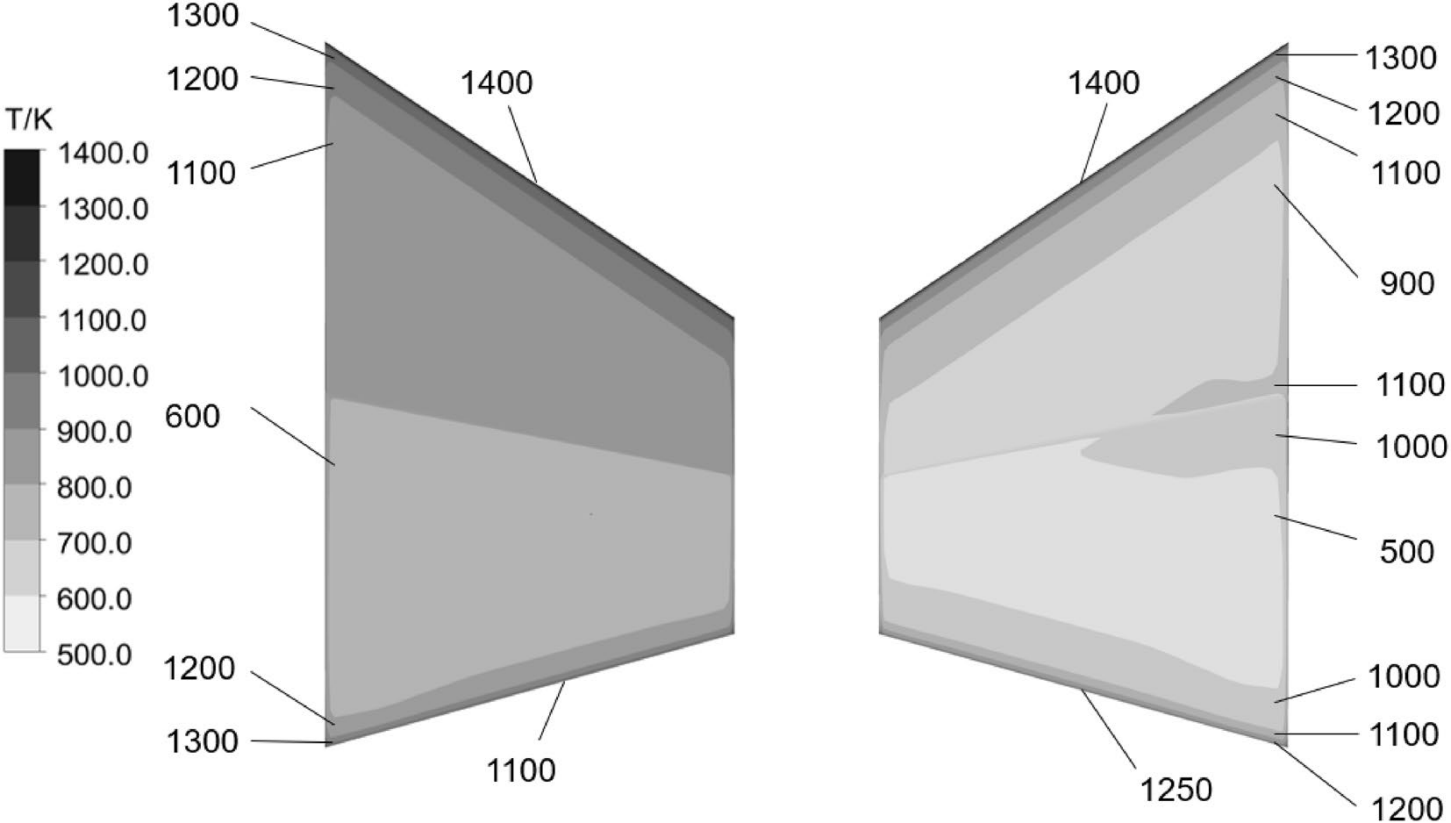
Temperature distribution of the upper surface (left) and lower surface (right).

Figure 17 shows the temperature distribution plot of the upper and lower surfaces of the wing at 50 s. From the plot, it can be observed that the temperature on the upper surface is significantly higher than that on the lower surface, with temperatures at the leading and trailing edges exceeding 1100 K. The temperature at the midpoints of the upper and lower surfaces is relatively lower, ranging from 500 to 1100 K.

### Thermal modal analysis of the wing

**Figure 18:**
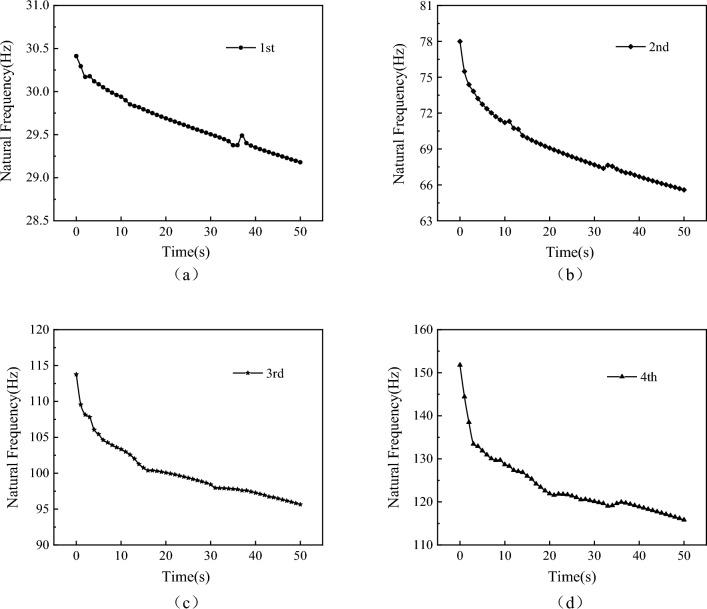
Time-varying curves of the first four modal frequencies.

Considering the influence of aerodynamic heating time, a pre-stressed modal analysis is performed on the wing with transient temperature field variations, calculating its natural frequencies and mode shapes, with the wing root subjected to fixed support boundary conditions. Figures (a), (b), (c), and (d) in Fig. 18 show the time variation curves of the first four thermal modal natural frequencies.

From the figures, it can be observed that the time variation patterns of the first four modes are generally similar. In the initial few seconds, the curves exhibit a relatively steep slope, indicating a rapid decrease in natural frequencies. Approximately 4 s later, the slope of the curves decreases, indicating a gradual slowdown in the variation of natural frequencies, yet without reaching equilibrium. The increase in temperature significantly affects the higher-order modal frequencies, leading to a reduction in the natural frequency differences among modes.

**Figure 19:**
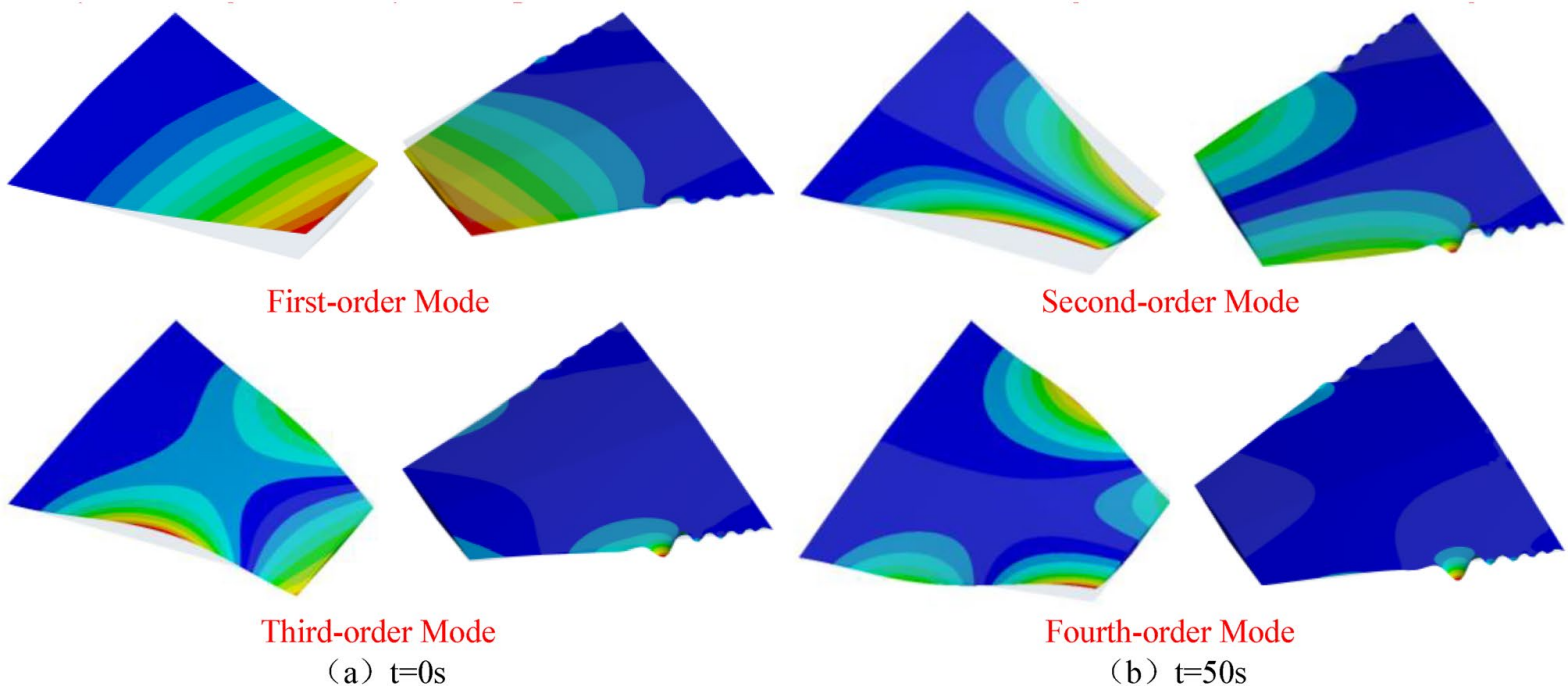
Compares the vibration modes of the first four modal shapes at different time instants.

Figure 19 presents a comparison of the first four thermal modal shapes at 0 and 50 s of aerodynamic heating time. Although the modal shapes of the first and second modes have changed, they can still be identified as the same mode; however, the third and fourth modes are significantly affected by localized modes, with modal shapes completely different from the initial 0-s mode. It can be observed from the figure that localized modal phenomena occur at the leading and trailing edge positions in the first four thermal modes. This phenomenon is primarily caused by the “softening” of wing structure stiffness. Figure 16 shows that at 50 s of aerodynamic heating, the temperatures at the leading and trailing edges of the wing reach 1400 K and 1150 K, respectively, while the temperature at the belly plate is only 700 K.

During hypersonic flight, the local temperature increase caused by aerodynamic heating may lead to thermal stress concentration in the wing material, with the local modes particularly pronounced at the leading and trailing edges. Furthermore, these local modes at the leading and trailing edges may subject the thermal protection system to higher temperatures and pressures, resulting in damage or failure of the thermal protection materials. The occurrence of local modes may also induce air vortices and turbulence, thereby affecting the aerodynamic performance of the aircraft, including variations in lift and drag.

**Figure 20:**
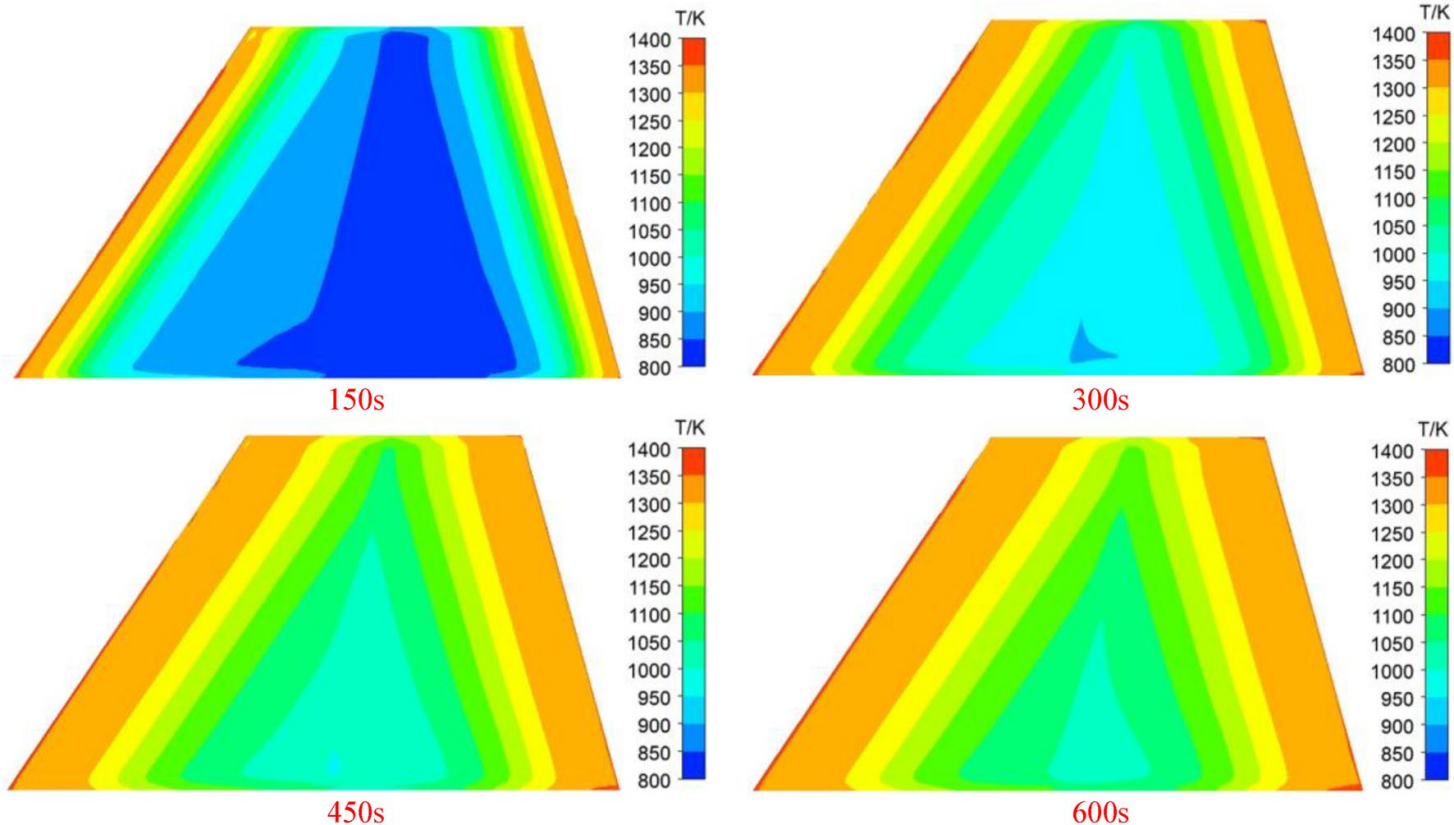
Temperature distribution of the wing surface at different times.

**Figure 21:**
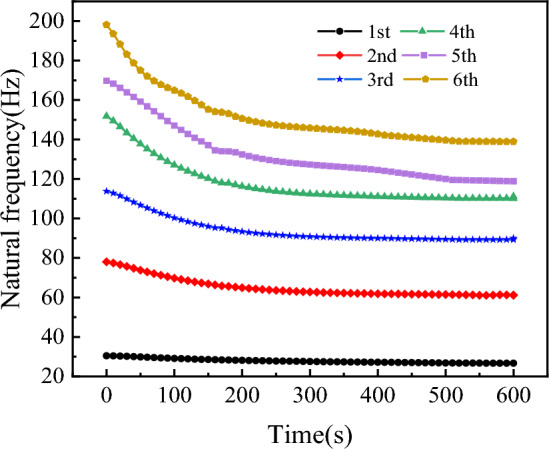
Time-varying curves of high-order modal frequencies.

The above calculation results indicate a significant influence of aerodynamic heating on high-order modes. Given that aerodynamic heating is a long-term process, investigating its effect on the frequencies of high-order modes becomes particularly important. Figure 20 illustrates the distribution of wing temperature at different instances, while Fig. 21 depicts the temporal variation curves of the fifth and sixth-order modal frequencies within 600 s.

From the figure, it can be observed that with the increase in heating time, the temperature at the belly plate position continues to rise, leading to the expansion of the high-temperature region in the wing structure. At the 600-s mark, the overall temperature of the wing has exceeded 950 K. The time-varying curve of the modal frequency gradually flattens after 500 s, indicating a converging trend. Specifically, due to changes in vibration shapes, the spacing between the fifth and fourth-order modal frequencies gradually decreases, potentially leading to coupled modal flutter phenomena in the wing.

### Analysis of the influence of stiffness on thermal modal characteristics

The influence of high temperatures on modal characteristics is primarily manifested in two aspects: material stiffness ${K}_{T}^{e}$ and additional geometric stiffness ${K}_{S}^{e}$. (a) High temperatures can alter material properties, leading to a reduction in the material stiffness ${K}_{T}^{e}$ of the wing due to the decrease in elastic modulus and the non-linear change in the coefficient of thermal expansion. (b) The increase in temperature induces temperature gradients in the wing, resulting in thermal stresses that cause additional geometric stiffness ${K}_{S}^{e}$ in the wing.

Considering the effects of ${K}_{T}^{e}$ and ${K}_{S}^{e}$ on thermal modal characteristics, this section establishes corresponding finite element models, calculates the time-dependent thermal modal variations under different influencing factors, and analyzes the impact of ${K}_{T}^{e}$ and ${K}_{S}^{e}$ on the first-order thermal modal at 46 s.

**Figure 22:**
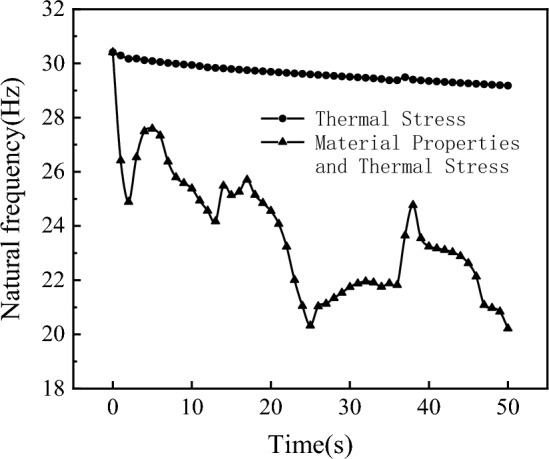
The time-varying curves of natural frequencies under different influencing factors.

Figure 22 depicts the time-dependent curve of the first-order wing mode. From the figure, it can be observed that considering only the influence of thermal stress results in a slow and essentially constant decrease in natural frequency. However, when considering the combined effects of thermal stress and material properties, the computed results are closer to reality. In the initial few seconds of intense shock wave heating, the wing rapidly heats up, and the wing experiences a “softening” phenomenon simultaneously with the decrease in material properties, causing a rapid decrease in natural frequency, with ${K}_{T}^{e}$ being the dominant factor. As thermal conduction progresses, the wing’s modal shapes change, and the increasing influence of additional stiffness caused by thermal stress becomes significant, even surpassing the impact of decreasing material properties, making ${K}_{S}^{e}$ gradually the dominant factor. Due to the large-scale time issue of aerothermal equilibrium, in this case, the dominant factors ${K}_{T}^{e}$ and ${K}_{S}^{e}$ alternately change, causing fluctuations in the natural frequency curve.

Table 4 presents an analysis of the impact of ${K}_{T}^{e}$ and ${K}_{S}^{e}$ factors on various modes at the heating time of 46 s, where 300 K denotes the mode at normal temperature. The results indicate that, compared to the normal temperature state, when considering only the influence of temperature rise on thermal modes, the impact of ${K}_{S}^{e}$ on thermal modes is relatively small, leading to computed results being larger than the actual values. Conversely, when considering only the effect of decreased material properties on thermal modes, the reduction in the material’s elastic modulus has a significant impact on thermal modes, resulting in computed results being smaller than the actual values.

**Table 4:** Analysis of thermal modal influences.

Mode number	300 K	${K}_{T}^{e}$	${K}_{S}^{e}$	${K}_{T}^{e}$ + ${K}_{S}^{e}$
1	30.412	15.689	29.246	22.134
2	77.99	40.233	66.01	57.269
3	113.76	58.685	96.318	91.904
4	151.78	78.298	117.13	101.17

## Conclusions

This paper utilizes the CFD/CSD method to calculate the steady-state temperature field for titanium alloy and C/SiC composite materials. Employing a segmented approach, the transient aerodynamic heating process of the wing is numerically simulated. The obtained aerodynamic heating is then used for thermal modal analysis, considering both thermal effects and material properties. The following conclusions can be drawn:

1. At Ma = 5, the leading edge of the wing exhibits the highest temperature, followed by the belly plate, and the trailing edge has the lowest temperature. Considering the combined effect of ${K}_{S}^{e}+{K}_{T}^{e}$, the stagnation temperature of C/SiC composite material can reach a maximum of 1600 K, which is below its usage limit. Therefore, C/SiC composite material can serve as an effective thermal protection material.
2. Under the intense influence of shock waves, the leading edge of the wing rapidly heats up to thermal equilibrium. During this process, the trailing edge temperature surpasses that of the belly plate. Following a slow heat conduction process, the temperature at the trailing edge gradually saturates, while the belly plate continues to experience ongoing heating. This paper simulates the transient aerodynamic heating process of the wing within the 0–600 s timeframe, and the overall structure of the wing does not reach thermal equilibrium.
3. With the increase in temperature, all first four modes exhibit a decreasing trend. Among them, the influence of temperature rise on higher-order modes is more pronounced. In the early stage of aerodynamic heating, temperature ${K}_{T}^{e}$ is the dominant factor, leading to a rapid decrease in frequency. As the heating time progresses, the thermal stress effect becomes prominent, and stiffness ${K}_{S}^{e}$ becomes the dominant factor, resulting in a gradual slowdown in the change of natural frequencies.
4. In the calculation process of wing thermal modes, considering only one factor not only deviates significantly from the actual values but also fails to accurately assess the aerodynamic-thermal and structural-thermal conduction processes. Therefore, when judging the frequency or mode changes of the aircraft by neglecting the impact of a certain factor, special caution should be exercised.

## Data Availability

All data generated or analyzed during this study are included in this published article.
